# A Miniaturized Transmitting LPDA Design for 2 MHz–30 MHz Uses

**DOI:** 10.3390/s21186034

**Published:** 2021-09-09

**Authors:** Wenjun Zhu, Lixin Guo

**Affiliations:** 1 Antenna Research and Develop Department, China Research Institute of Radiowave Propagation, Qingdao 266107, China; hanks188@126.com; 2 Physics and Optoelectronic Engineering School, Xidian University, Xi’an 710071, China

**Keywords:** antenna miniaturization, high frequency, transmitting antenna

## Abstract

A miniaturized horizontal polarized high frequency transmitting LPDA is presented. In use of the dipole transformation and antenna coefficient optimization methods, a 65% reduction in the size was achieved with the electrical performance kept in a competitive level. Full-wave simulation results showed a stable directional pattern and lower VSWR over the impedance bandwidth of 2 to 30 MHz. The gain bandwidth can reach the range of 4–30 MHz, meanwhile, there is only minor degradation on gain in frequencies under 4 MHz.

## 1. Introduction

To meet the increasing demand for miniaturized antenna, researchers developed all sorts of miniaturization technologies. These techniques have reduced the size of the antenna to some extent [1,2,3]. However, not all the technologies were appropriate for high frequency (HF) antennas. Most of the HF antennas were too large to apply the techniques, such as magneto-dielectric ferrite materials [4], as well as having a complicated structural approach [5,6,7]. Researchers in this field tend to seek more economical and practical methods to reduce the HF antennas’ size. Structural transformation [8,9,10] and wideband loading technology [11] were effective approaches in recent years. A helical structure was proved to be valid for antenna miniaturization in the literature [12,13]; the helical form can reduce the antenna size to some extent. However, the drawbacks of lower gain and limited communication range restrict its use fields. Besides, the complicated helical structure is harder to create than a simulation. Machining accuracy has great influence on the electrical performance. To broaden the structural bandwidth, some helical antennas with a complex network for a sectional match can only be applied in the receiving system. Characteristic theory was applied to the platform-mounted HF antenna design and miniaturization [14,15,16,17]. Limited by its operating environments, the whole HF band cannot be covered by this kind of antenna. Beside this, authors in [18] proposed a wire duoconical monopole antenna design that had a bandwidth of 7.5–25 MHz. The gain was above 0.2 dBi. The bandwidth had not covered the lower frequencies in the HF band and a higher gain would be more appropriate for wake signal receiving. According to the ITU P.372-14 [19], most of the noises in the HF band are vertically polarized, so that the monopole form is an adverse condition in HF band communication. Owing to their constant radiation characteristics over broad bandwidth, a Log-Periodic Dipole Antenna (LPDA) was widely used in the HF band. Meanwhile, when the LPDA needed to cover the entire short wave frequency band (2–30 MHz), the antenna size usually had to be designed very large to fit the lower frequency requirement. This disadvantage enhanced the construction difficulty and increased the floor space. Miniaturization became an important issue for LPDA in modern-day HF communication.

**Figure 1:**
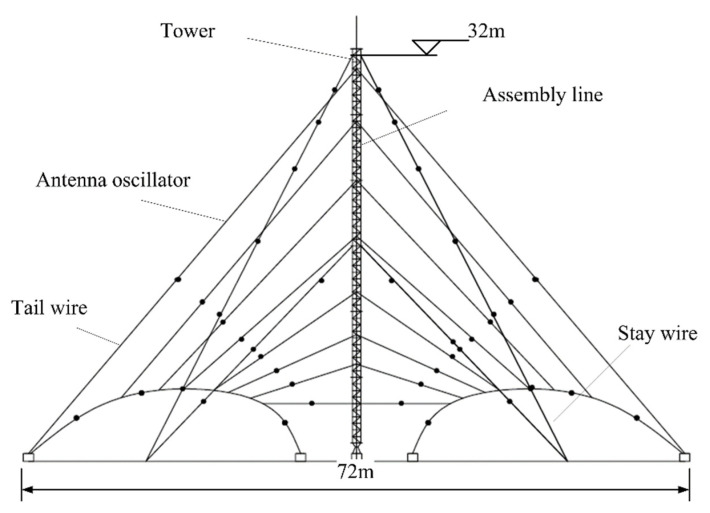
TCI-530 LPDA.

There has been some research on HF LPDA miniaturization in recent years. In use of antenna structure transformation, authors in [20] presented a miniaturized invert-V LPDA design worked in 6–30 MHz. Its bandwidth was still not broad enough, though the miniaturized antenna has a good performance in 6–30 MHz. Applying to the same miniaturize principle as [20], the American TCI corporation produced one type of horizontal polarized HF LPDA with a bandwidth of 2–30 MHz, TCI-530 [21]. As is illustrated in Figure 1, it was 32 m high and 70 m wide.

In this paper, a novel miniaturized design of LPDA was proposed, which was inspired by the antenna that was shown in Figure 1. A 65% reduction in the size was achieved without any obvious impedance change over the operation frequency range of 2–30 MHz. The gain characteristics were maintained on a good level over 4–30 MHz. The antenna could be employed in both of the receiving and transmitting systems. In transmitting systems, it has a 5 kW power endurance capability. As a deformation form of the regular LPDA, our proposal has a similar pattern feature. The patterns kept constant over the entire bandwidth and the maximum 3 dB radiation angle on the vertical plane was nearly 30–60°; combined with the characteristics of ionosphere and the information channel, the antenna has a communication range of 0–500 km that can be utilized in navigation, broadcast, signal monitoring, etc.

A comprehensive comparison of our proposal and other antennas that work in the HF band is demonstrated in Table 1. Compared with the antennas proposed in the literature [12,13,14,15,16,17,18], our design has a broader impedance bandwidth that covers nearly the entire HF band (2–30 MHz). The radiation patterns are preferable to others for their stability. The horizontal polarization and transmit–receive usability ensured wider application of our proposal. Amongst the antennas presented in recent literatures, our proposal and TCI 530 have a broader bandwidth and more stable performance over the whole operation band for their frequency independent structure. Other smaller antennas had a narrower bandwidth and lower gain when the total sizes were reduced.

In this paper, a novel miniaturized LPDA design employing the combination method of element deformation and coefficient variation was presented. The proposal effectively reduced the antenna size without drawbacks on its performance. The rest of this paper is arranged as follows: in Section 2 we presented the miniaturization design and optimization. Simulation and verification were shown in Section 3. Finally, we draw conclusions in Section 4.

## 2. Miniaturization Design and Optimization

Compared with the conventional full range HF horizontal polarized LPDA that was over 100 m long, the antenna showed in Figure 1 had been miniaturized by its invert-V structure that reduced the horizontal occupation area. It is a known fact that the entire size of a full range HF LPDA is mostly defined by its lower frequency dipole elements. As shown in Figure 1, the longest dipole element defined the height and width of the antenna. To make the antenna’s structure sustainable and wind-resistant, the structural items such as stay wires and tail wires must be connected to the antenna and the support tower accordingly. This made the antenna appear even larger. Hence the miniaturization procedure should be carried out in two aspects: electrical and structural.

### 2.1. Electrical Miniaturization and Antenna Design

**Figure 2:**
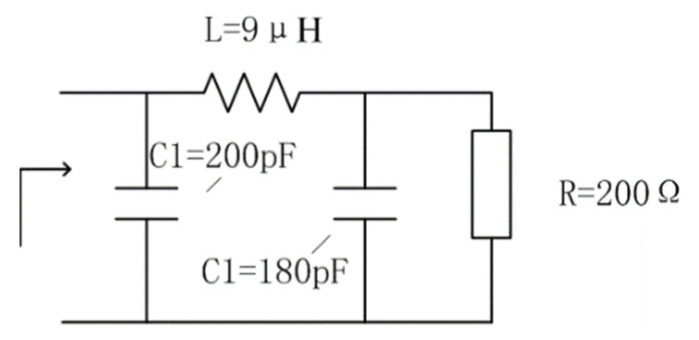
The longest dipole of the miniaturized LPDA.

In the antenna theory of LPDA, the cutoff frequencies of the truncated structure can be determined by the electrical lengths of the elements’ structure [3]. Different dipoles were corresponding to different operation frequencies. The longest dipoles were responding to the lower frequencies. Therefore, we can reduce the LPDA size by transformation of the longest dipole, inspired by methods presented in [3,5,22,23], where the shape of the longest dipole was changed and loaded with networks. Herein the longest dipole elements were changed from a single wire into three wires and a loaded network in our approach. The external form and the network is shown in Figure 2; these three wires were in a same plane and half of the transformed dipole was 17 m long; the diameter of the wire was 6 mm, the width of the three wire plane dipole was 1.5 m. The network consisted of a series of inductors and a capacitor with their detailed value showed in Figure 2. It was parallel connected with the assembly line and mainly operated in 2–4 MHz. Moreover, due to the attenuation involved by the network, there was degradation of efficiencies in 2–4 MHz; consequently, the gain bandwidth can only reach 4–30 MHz.

**Figure 3:**
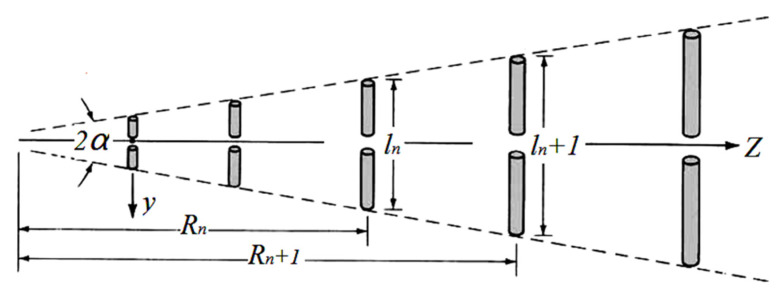
The log-periodic dipole array.

However, only modification on the longest dipole cannot reduce the antenna size to an ideal level. Therefore the method of variable coefficients was applied in our proposal to achieve further miniaturization. The LPDA coefficients σ and *τ* had been optimized according to the antenna theory. Here σ is the spacing factor and *τ* is the proportionality factor. The basic coefficients *τ* σ and their relationships were determined by (1) [24]. As is shown in Figure 3, *R* is the dipole spacing and l the length. Typical method of Carrel was used for reference in the design procedure [24]. The procedure can be divided into five steps as is shown below.

(1) Given D_0_ (dB), determine σ and τ from figure of computed contours of constant directivity versus σ and *τ* for log-periodic dipole arrays in [24]. Here the given gain D_0_ was set as 5 dB and *σ* = 0.05, *τ* = 0.84;
(2) Determine the active region bandwidth *B_ar_* and designed bandwidth *B_s_* by formula (2). The desired bandwidth was 4–30 MHz;
(3) Find the total length of the structure L and the number of elements N in use of (3);
(4) Determine the average characteristic impedance of the elements Z_a_ and the characteristic impedance of the feeder line Z_0_;
(5) Optimize *σ* and *τ* with the assistant of full-wave simulation software FEKO.

The main coefficients were determined as follows: *σ* = 0.05, *τ* = 0.84 and the number of dipole elements were 16. A LPDA with varying factors of *σ* and *τ* for each dipole elements would have miniaturized size and competitive electrical performance compared with the conventional antenna [25]. Coefficients of each element were optimized in consideration of the size and the electrical performance with the assistance of the full-wave simulation software EMSS FEKO by a sort of simulation loop.

(1) $$\tau =\frac{R_{n}}{R_{n+1}}\;,\sigma =\frac{R_{n+1-}R_{n}}{2l_{n+1}},\;\alpha =\tan^{-1}\left[\frac{1-\tau }{4\sigma }\right]$$

(2) $$B_{\mathrm{ar}}=1.1+7.7{\left(1-\tau \right)}^{2}cos\alpha ,\;B_{s}=\tau^{1-N}$$

(3) $$L=\frac{\lambda_{max}}{4}\left(1-\frac{1}{B_{s}}\right)cot\alpha ,\;N=1+\frac{ln(B_{s})}{ln\left(\frac{1}{\tau }\right)}$$

Besides, the included angles, *θ* between the two arms of each dipole in the LPDA will affect the electrical performance and the size. The best value of each angle should balance both of the electrical and structural needs. The full-wave simulation software EMSS FEKO was applied to simulate and optimize our design. The optimized parameters are given in Table 2. Besides the deformed longest element, the antenna consisted of 16 pairs of dipoles and a set of assembly lines. The output impedance was 200 Ω so that a 4:1 impedance balun was fitted to the feed point. Designed antenna coefficients are given in Table 2. The total length L of the antenna was 21.15 m; the shortest dipole was 3.8 m. The diameter of the dipoles was 6 and 8 mm for the assembly line.

### 2.2. Structural Miniaturization

**Figure 4:**
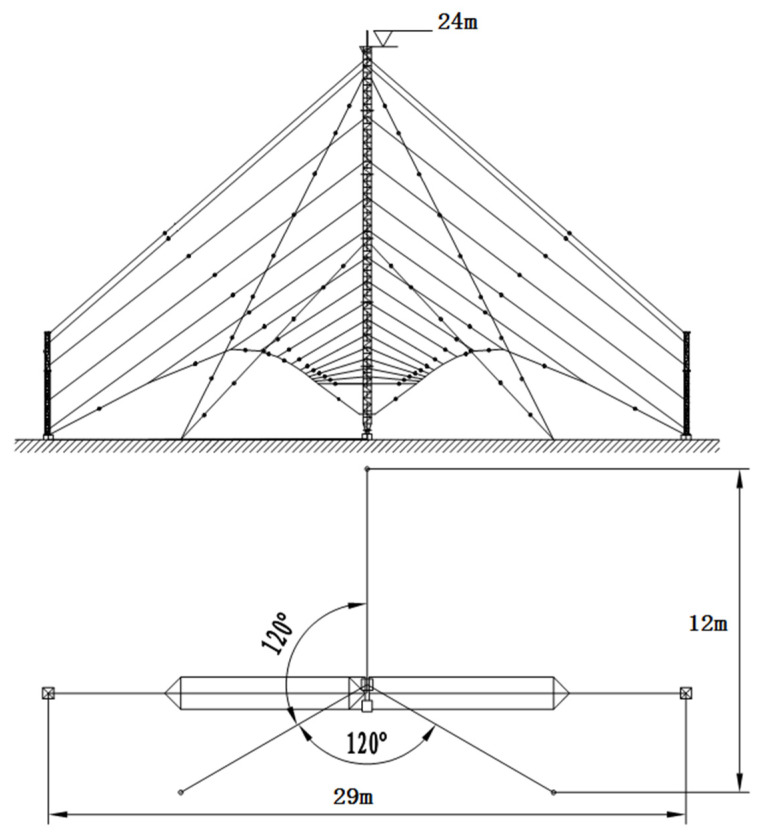
Shape of the miniaturized antenna.

The obvious difference between the HF antennas and other antennas that work in a higher frequency happens to the gigantic structure. Most of the HF antennas need supporting items or stay wires to obtain their electrical size and resist the strong wind in the outdoor circumstance. The proposal of our designed LPDA is shown in Figure 4; through comparisons of Figure 1 and Figure 4, one can discover that the necessary structural components take a considerable part of the entire antenna size. Therefore, the occupational size could be miniaturized if we adopt a smaller but sustainable structure.

In our proposal, the tail wires on both sides of the antenna were substituted for a pair of insulating bar with stay wires. As is illustrated in Figure 4, the antenna height was miniaturized from 32 m to less than 24 m; the horizontal size was shrunk from 72 to 24 m. Even the horizontal projection was a part no longer than 30 m. Finally, the total front size of the antenna was reduced by 65%.

## 3. Simulation and Verification

**Figure 5:**
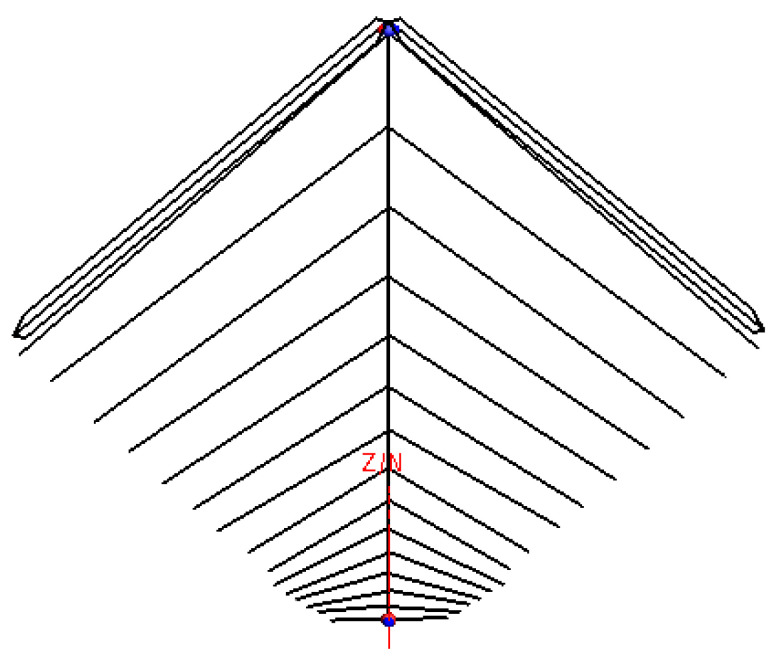
The simulation model.

With the aid of EMSS FEKO, a full-wave electromagnetic simulation was carried out to evaluate the performance of our proposal and the former antenna. As can be observed in Figure 5, 524 metallic wire segments were used to create the model’s electrical structure. Exact Sommerfeld integrals were utilized to calculate the ground influence. Ground coefficients were set at a relative permittivity of *ε_r_* =15 and a conductivity of *σ* = 0.01.

### 3.1. VSWR Results

**Figure 6:**
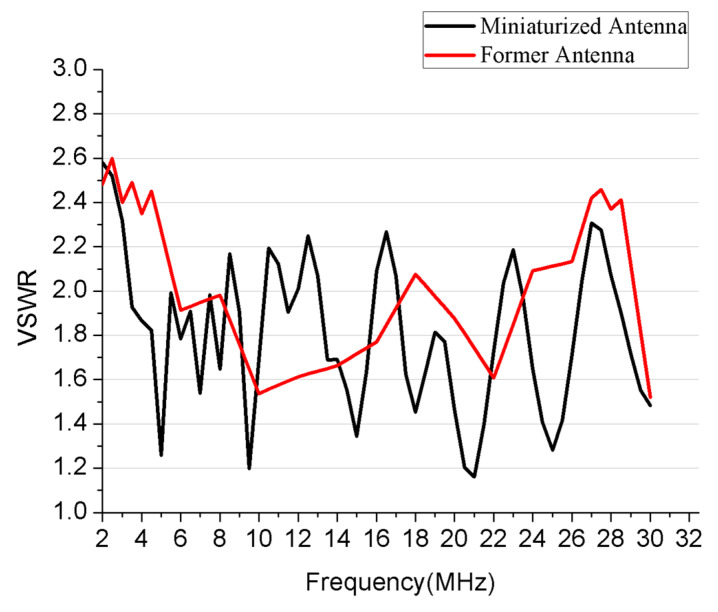
Simulated VSWR of the former antenna and the miniaturized one.

It is known as a fact that when the voltage standing wave ratio (VSWR) equals to three, only 25% of the power will be lost. Hence 2.5 was commonly set as an acceptable value for the VSWR of HF antenna. The comparison of simulated VSWR of our proposal and the former antenna was shown in Figure 6. The results indicated that the VSWR of the miniaturized antenna has similar values that are lower than 2.6. Most of the VSWR were close to 2.2 or lower. Our proposal had a good VSWR performance over the broad bandwidth. This indicates that the antenna is appropriate for transmitting.

### 3.2. Simulated Pattern

**Figure 7:**
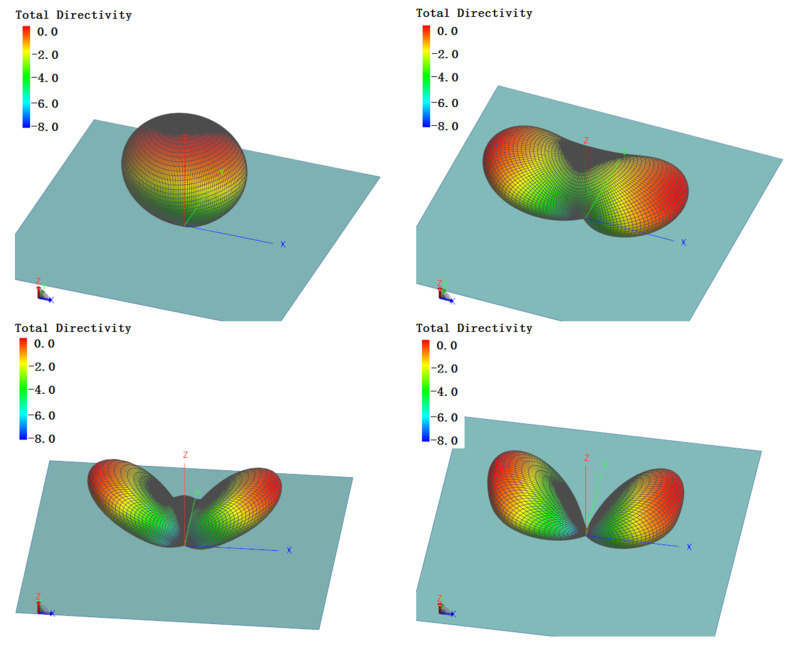
3D pattern of the miniaturized antenna.

**Figure 8:**
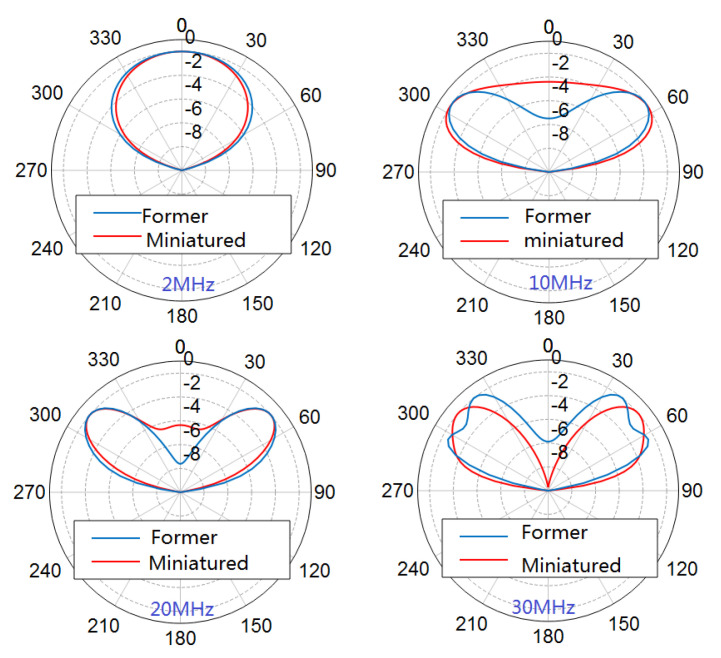
Directivity pattern comparison.

The simulated 3D pattern is shown in Figure 7; it can be seen that miniaturization has not caused variations in patterns. The comparison of normalized directivity patterns of the former antenna and the miniaturized one is shown in Figure 8. There have been no significant changes in patterns of the miniaturized antenna in the entire bandwidth. The stability of the radiation pattern was proved by the simulation. The radiation angle indicates that our proposal was propitious for short and middle range communication.

### 3.3. Simulated Gain

**Figure 9:**
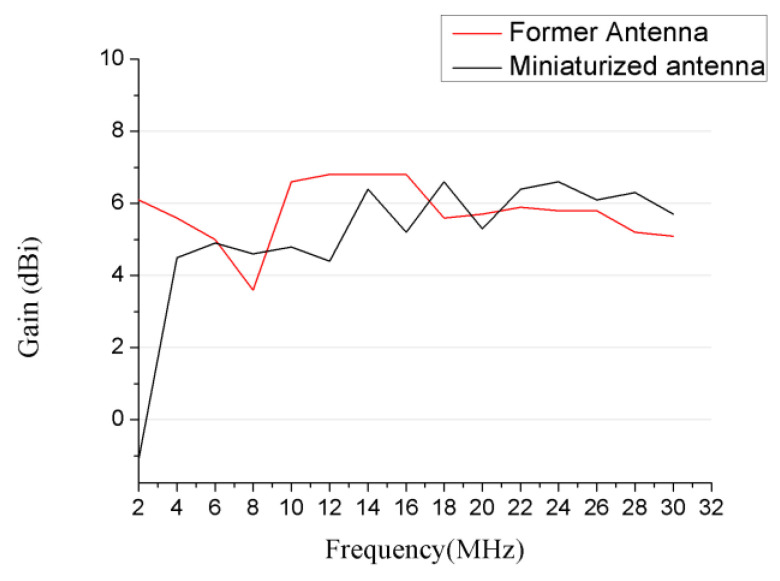
Simulated gain.

As is shown in Figure 9, the simulated gain of our proposed LPDA has a similar value in comparison with the former antenna in operation frequency range over 4 MHz. Being affected by the added network on the longest dipole, the antenna efficiency was decreased in frequencies under 4 MHz; as a result, the gain was consequently decreased in the same frequency.

It can be observed from Figure 6 that the miniaturized antenna had a similar electrical performance in comparison to the former one. Besides, a comparison of our proposal and the former antenna was listed in Table 3 as follows. Most of the conventional HF antennas that work in the same bandwidth range from 2 to 30 MHz cannot achieve the same level of electrical performance.

### 3.4. Verification

**Figure 10:**
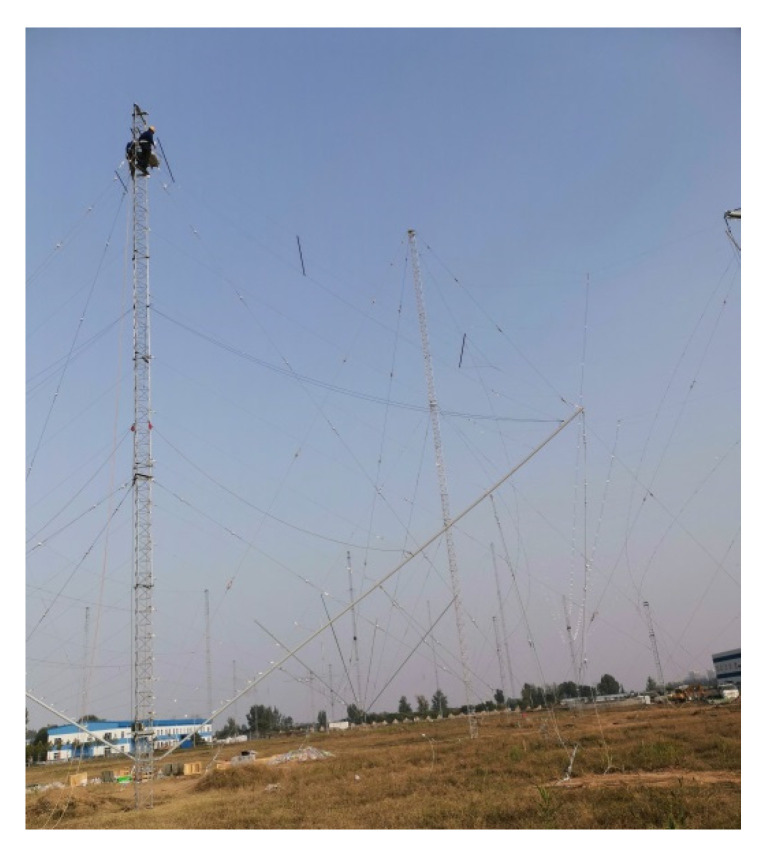
Fabricated miniaturized transmitting LPDA.

**Figure 11:**
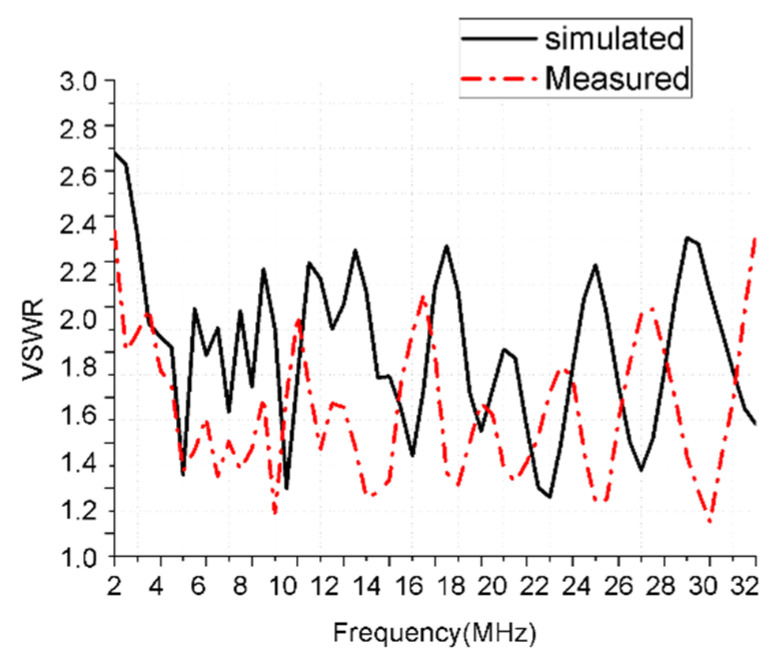
Tested VSWR and simulated VSWR.

Photographs of fabricated antennas are shown in Figure 10. The practical VSWR was measured and the comparison with the simulation was shown in Figure 11. Power endurance capacity was also tested and proved by an experiment. A set of 7/8” coaxial cables and a 5 kW transmitter were used to test the power endurance of the miniaturized antenna. Three frequencies were operated during the experiment procedure, which were 5.5, 15.4, and 25.5 MHz. Each operating frequency had been worked for one hour with the carrier wave type of CW (continuous wave) on maximum power output. The antenna had a stable performance during the power endurance test.

## 4. Conclusions

A miniaturized transmitting LPDA design based on the combination of loading and varying parameters was presented and optimized in this paper. The operating frequency range was 2–30 MHz. The optimization of the support structure obtained further miniaturization. Compared with the antenna in the same style, the optimized antenna was expected to have similar VSWR and gain with the conventional LPDA while its size was miniaturized by 65%, and the gain and pattern were verified by simulation. The antenna was fabricated and the VSWR was tested. Besides, the performance of 2~4 MHz can be further optimized, considering the available designs present in the literature; more complicated dipole forms such as helixes and more effective match networks for lower frequencies will be studied in the subsequent work.

## Figures and Tables

**Table 1:** Comparison of our proposal and the other antennas.

Item	Our Proposal	TCI 530	Duoconical Monopole	Invert-V LPDA
Dimension	29 m × 24 m	72 m × 32 m	20 m × 6 m	30 m × 19.8 m
Impedance Bandwidth	2–30 MHz	2–30 MHz	7.5–25 MHz	6–30 MHz
Gain	5–7 dBi (over 4 MHz)	4–7 dBi	0.2 dBi	2–8 dBi
VSWR	≤2.5	≤2.5	≤3.0	≤2.5

**Table 2:** Coefficients of the designed LPDA.

Dipole Number	*Σ*	*τ*	*Θ* (°)
1	0.050	0.86	93
2	0.051	0.86	102
3	0.053	0.86	104
4	0.054	0.86	110
5	0.055	0.86	112
6	0.057	0.87	116
7	0.058	0.87	118
8	0.059	0.87	123
9	0.060	0.87	130
10	0.060	0.87	135
11	0.060	0.88	140
12	0.060	0.88	145
13	0.061	0.88	155
14	0.061	0.88	163
15	0.062	0.88	175
16	0.062	0.88	180

**Table 3:** Comparison of our proposal and the former antenna.

Item		Our Proposal	Former Antenna
Dimension		29 × 24 m	72 × 32 m
Impedance Bandwidth		2–30 MHz	2–30 MHz
Gain		5–7 dBi (over 4 MHz)	4–7 dBi
VSWR		≤2.5	≤2.5
Maximum Radiation Angle	2 MHz	0°	0°
	15 MHz	60°	57°
	30 MHz	50°	48°
3 dB Lobe Width (Horizontal plane)	2 MHz	102°	107°
	15 MHz	86°	80°
	30 MHz	83°	79°

## Data Availability

The data presented in this study are available on request from the corresponding author.
